# Roux-en-Y and Billroth II Reconstruction after Pancreaticoduodenectomy: A Meta-Analysis of Complications

**DOI:** 10.1155/2020/6131968

**Published:** 2020-12-04

**Authors:** Fulin Ma, Yong Fan, Lina Zhang, Zhiqiang Zhao, Yuanhua Nie, Minxue Chen, Chen Wang

**Affiliations:** ^1^The Second Clinical Medical College of Lanzhou University, Lanzhou 730030, China; ^2^Department of General Surgery, Lanzhou University Second Hospital, Lanzhou 730030, China

## Abstract

**Objective:**

To evaluate Roux-en-Y and Billroth II reconstruction following pancreaticoduodenectomy (PD).

**Methods:**

PubMed, Embase, the Cochrane Library, and the Web of Science were searched to identify randomized controlled trials (RCTs) and controlled clinical trials that compared Roux-en-Y and Billroth II reconstruction following PD up to December 2019. RevMan 5.3 software was used for the statistical analysis.

**Results:**

Four RCTs and five controlled clinical trials were included, with a total of 1,072 patients (500 and 572 patients in the Roux-en-Y and Billroth II groups, respectively). No significant differences in delayed gastric emptying (DGE), A-grade DGE, B-grade DGE, or C-grade DGE were observed between the Roux-en-Y and Billroth II reconstruction groups after PD (odds ratio [OR] = 1.01, 95% confidence interval [CI]: 0.50–2.03, *P* = 0.98; OR = 0.49, 95% CI: 0.17–1.45, *P* = 0.20; OR = 0.63, 95% CI: 0.29–1.38, *P* = 0.25; and OR = 2.13, 95% CI: 0.38–11.99, *P* = 0.39). No significant difference in the incidence of postoperative pancreatic fistula, abscess, bile leaks, infection, postoperative bleeding, or the length of the postoperative hospital stay was observed between the Roux-en-Y and Billroth II groups (*P* > 0.05), but the operation time was significantly different (mean difference [MD] = 31.65, 95% CI: 7.14–56.17, *P* = 0.01).

**Conclusions:**

Billroth II reconstruction after PD did not significantly reduce the incidence of DGE or other complications but shortened the operation time compared to Roux-en-Y reconstruction. However, the results must be verified by further high-quality, large RCTs or controlled clinical trials.

## 1. Introduction

The incidence of pancreatic cancer is rising, and it is estimated that pancreatic cancer will become the second most deadly cancer in the world by 2030 [1]. Scholars have performed a considerable amount of research on pancreaticoduodenectomy (PD) since Whipple et al. first proposed the concept in 1935 [2]. Complications after PD have a significant impact on the postoperative quality of life of patients, for whom the total and clinically relevant delayed gastric emptying (DGE) incidence rates are 27.7% and 14.3%, respectively. The incidence of DGE is associated with the pancreatic resection type, pylorus preservation status (yes or no), antecolic and retrocolic gastrojejunal anastomosis, and gastrojejunal anastomosis type [3]. The incidence of DGE after Billroth II and Roux-en-Y gastrojejunal reconstruction has remained controversial. Based on the hypothesis that Roux-en-Y anatomy can prevent gastric contents from activating trypsin, Machado et al. [4] proposed that Roux-en-Y “protects” the pancreatojejunostomy. Results of single-center randomized controlled trials (RCTs) comparing the incidence of DGE between Roux-en-Y and Billroth II reconstruction are not consistent [5–8]. Meta-analyses have also reported contrasting results. Yang et al. [9] proposed that Billroth II reconstruction reduces the incidence of clinical DGE compared to Roux-en-Y; however, Li et al. [10] reported no difference between the two reconstruction types.

The purpose of this study is to evaluate the ability of Roux-en-Y and Billroth II reconstruction following PD to prevent DGE and other complications.

## 2. Materials and Methods

### 2.1. Selection Criteria

#### 2.1.1. Inclusion Criteria

The inclusion criteria are as follows: (1) a clinical comparative study between Roux-en-Y and Billroth II gastrojejunal reconstruction following PD, (2) RCT or clinical controlled trial (CCT), and (3) English.

#### 2.1.2. Exclusion Criteria

The exclusion criteria are as follows: (1) no outcome indicators, (2) pancreaticogastrostomy, (3) reviews and case reports, and (4) gastrectomy history.

### 2.2. Search Strategy and Screening Methods

PubMed, Embase, the Cochrane Library, and the Web of Science were searched up to December 2019. The search terms were as follows: ((((((pancreatoduodenectomy) OR pancreaticoduodenectomy) OR Whipple))AND(((Roux-en-Y) OR double loop) OR dual loop))AND((((Billroth II) OR conventional reconstruction) OR conventional loop reconstruction) OR single loop))AND(((delayed gastric emptying) OR pancreatic fistula) OR postoperative pancreatic fistula). Published RCTs and CCTs comparing Roux-en-Y and Billroth II reconstruction following PD were searched. Two researchers independently screened the studies, cross-checked their quality, and asked a third researcher to settle any controversies regarding whether to include a study.

### 2.3. Quality Assessment

The modified Jadad Scale [11] and Newcastle-Ottawa Scale [12] were used to assess the quality of the RCTs and CCTs, respectively.

### 2.4. Data Extraction

The general information extracted included the first author's name, year of publication, number of patients included in the study, their age and gender, and the study period, study type, and country. The primary outcomes were DGE, A-grade DGE, B-grade DGE, and C-grade DGE, and the secondary outcomes were postoperative pancreatic fistula (POPF), abscess, bile leak, infection, postoperative bleeding, operation time, and length of postoperative hospital stay.

### 2.5. Statistical Analysis

RevMan 5.3 software (Cochrane Collaboration, Copenhagen, Denmark) was used for the statistical analysis. Odds ratio (OR), mean difference (MD), and 95% confidence interval (CI) data were generated, and differences were considered significant when *P* < 0.05. Regarding heterogeneity assessment, a fixed-effects model was used when *P* > 0.1 and *I*^2^ < 50%, and a random-effects model was used when *P* < 0.1 and *I*^2^ > 50%. Subgroup analyses were performed when *P* < 0.1 and *I*^2^ > 50% to determine the reason for the heterogeneity. We prepared funnel plots to assess publication bias.

## 3. Results

### 3.1. Literature Search Results

**Figure 1:**
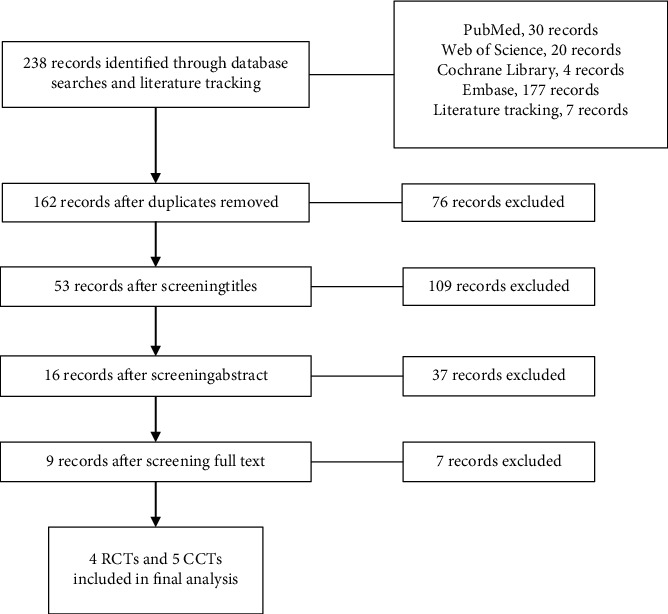
PRISMA flow chart of the literature screening process.

Ultimately, nine high-quality articles [5–8, 13–17], including four RCTs [5–8] and five CCTs [13–17], were included. The literature screening process is shown in Figure 1. The general parameters of the included studies are shown in Table 1, and the pathologies, surgery-related parameters, and quality scores of the included studies are shown in Table 2.

### 3.2. Primary Outcomes

**Figure 2:**
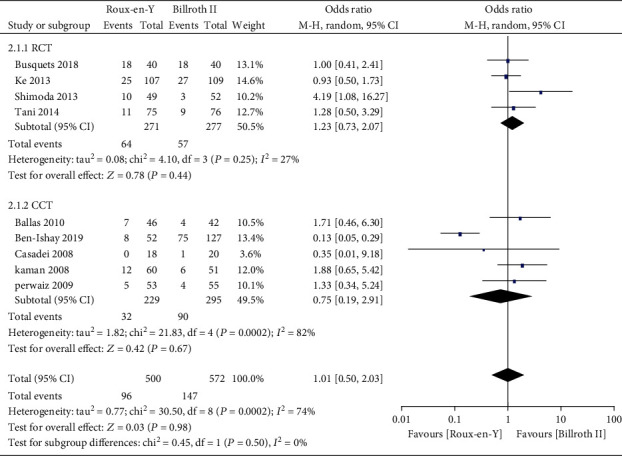
Forest plot of the incidence of delayed gastric emptying (DGE).

**Figure 3:**
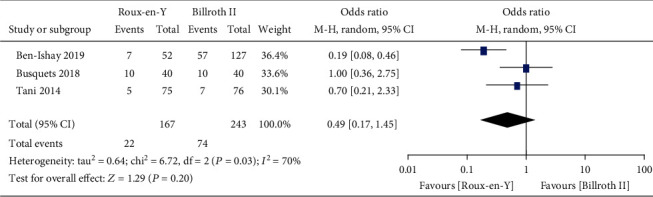
Forest plot of the incidence of A-grade delayed gastric emptying (DGE).

**Figure 4:**
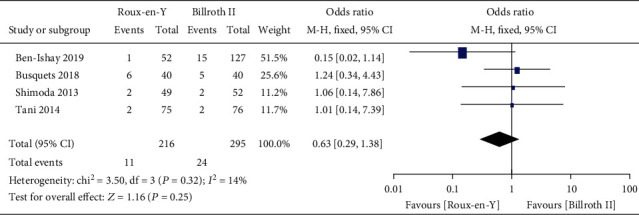
Forest plot of the incidence of B-grade delayed gastric emptying (DGE).

**Figure 5:**
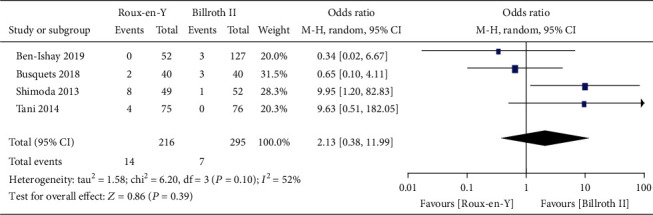
Forest plot of the incidence of C-grade delayed gastric emptying (DGE).

Nine studies [5–8, 13–17] provided the incidence of DGE, three [6, 7, 16] provided the incidence of A-grade DGE, four [5–7, 16] provided the incidence of B-grade DGE, and four [5–7, 16] provided the incidence of C-grade DGE. The heterogeneity results were *P* = 0.0002, *I*^2^ = 74%; *P* = 0.03, *I*^2^ = 70%; *P* = 0.32, *I*^2^ = 14%; and *P* = 0.10, *I*^2^ = 52%, respectively, as shown in Figures 2–5. The fixed-effects model was selected for B-grade DGE, while the random-effects model was used for the other outcomes. The overall effect sizes of the above outcomes were as follows: OR = 1.01, 95% CI: 0.50–2.03; OR = 0.49, 95% CI: 0.17–1.45; OR = 0.63, 95% CI: 0.29–1.38; and OR = 2.13, 95% CI: 0.38–11.99, respectively. These results suggest that the outcomes were not significantly different (*P* > 0.05) between the Roux-en-Y and Billroth II groups.

### 3.3. Secondary Outcomes

**Figure 6:**
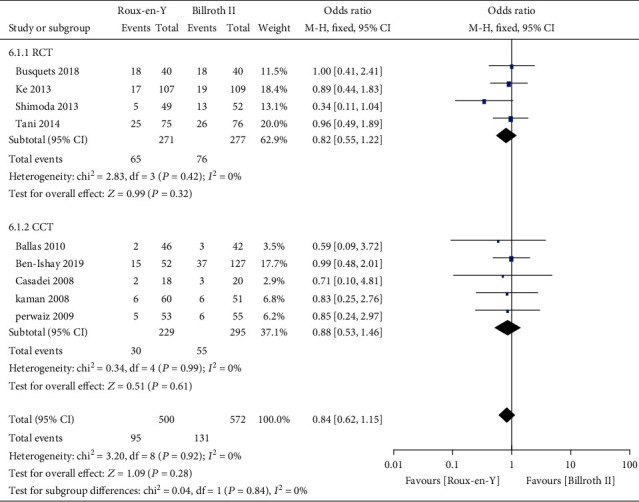
Forest plot of the incidence of postoperative pancreatic fistula (POPF).

**Figure 7:**
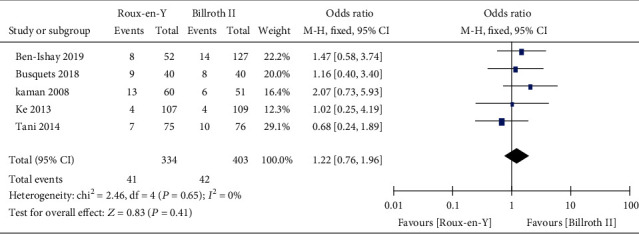
Forest plot of the incidence of abscess.

**Figure 8:**
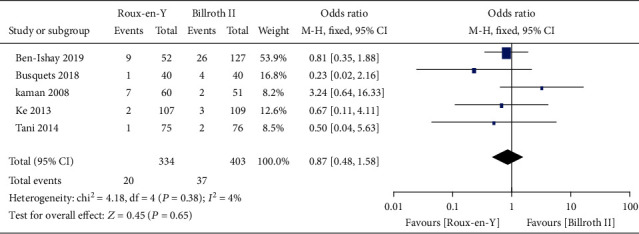
Forest plot of the incidence of bile leak.

**Figure 9:**
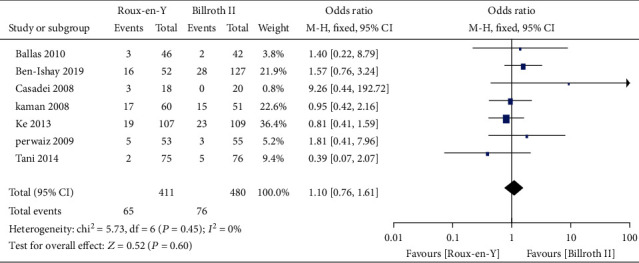
Forest plot of the incidence of infection.

**Figure 10:**
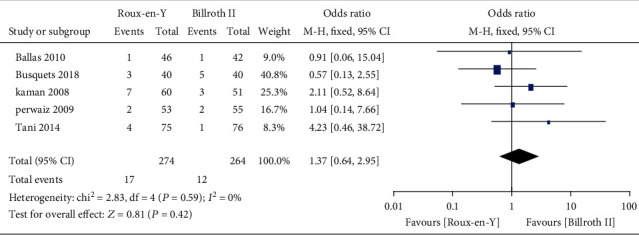
Forest plot of the incidence of postoperative bleeding.

**Figure 11:**
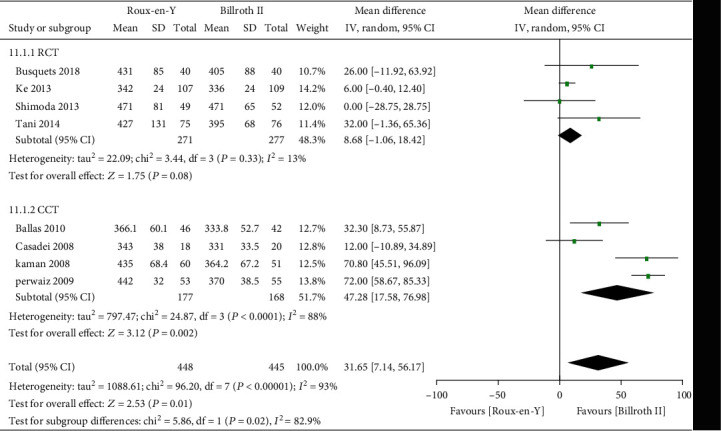
Forest plot of operation time.

**Figure 12:**
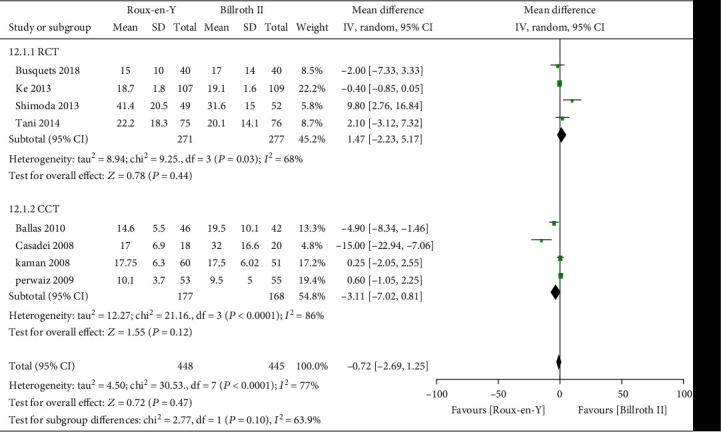
Forest plot of the length of postoperative hospital stay.

Nine studies [5–8, 13–17] provided the incidence of POPF, five [6–8, 14, 16] provided the incidence of abscess, five [6–8, 14, 16] provided the incidence of bile leaks, seven [7, 8, 13–17] provided the incidence of infection, five [6, 7, 13–15] provided the incidence of postoperative bleeding, eight [5–8, 13–15, 17] provided the operation time, and eight [5–8, 13–15, 17] provided the length of postoperative hospital stay. The heterogeneity test results were *P* = 0.92, *I*^2^ = 0%; *P* = 0.65, *I*^2^ = 0%; *P* = 0.38, *I*^2^ = 4%; *P* = 0.45, *I*^2^ = 0%; *P* = 0.59, *I*^2^ = 0%; *P* < 0.00001, *I*^2^ = 93%; and *P* < 0.0001, *I*^2^ = 77%, respectively, as shown in Figures 6–12. The random-effects model was used to analyze the length of operation and postoperative hospital stay data; for analysis of the other outcomes, the fixed-effects model was used. The overall effect sizes of the above outcomes were as follows: OR = 0.84, 95% CI: 0.62–1.15; OR = 1.22, 95% CI: 0.76–1.96; OR = 0.87, 95% CI: 0.48–1.58; OR = 1.10, 95% CI: 0.76–1.61; OR = 1.37, 95% CI: 0.64–2.95; MD = 31.65, 95% CI: 7.14–56.17; and MD = −0.72, 95% CI: −2.69–1.25, respectively. The results suggested no significant differences in outcomes (all *P* > 0.05) between the Roux-en-Y and Billroth groups, except for operation time (*P* < 0.05).

### 3.4. Sensitivity and Subgroup Analyses

The heterogeneity data for DGE were *P* = 0.0002, *I*^2^ = 74%; the results did not change after the subgroup analysis, indicating high reliability thereof. The heterogeneity values of A-grade DGE and C-grade DGE were *P* = 0.03, *I*^2^ = 70%, and *P* = 0.10, *I*^2^ = 52%, respectively. We eliminated each study one by one, and the results showed good stability. The heterogeneity data for operation time and length of postoperative hospital stay were *P* < 0.00001, *I*^2^ = 93%, and *P* < 0.0001, *I*^2^ = 77%, respectively. We conducted a subgroup analysis based on the RCT and CCT groups. Operation time in the RCT group was not significantly different between the two reconstructions, while in the CCT group Roux-en-Y reconstruction had a longer operation time (MD = 47.28, 95% CI: 17.58–76.98, *P* = 0.002). Subgroup analysis did not reveal a significant difference in length of postoperative hospital stay between the Roux-en-Y group and Billroth II groups (*P* = 0.44 and *P* = 0.12 for RCT and CCT, respectively). Therefore, operation time was associated with study type. A high-quality RCT or CCT is still needed to verify the operation time difference between the two reconstructions.

### 3.5. Bias Analysis

**Figure 13:**
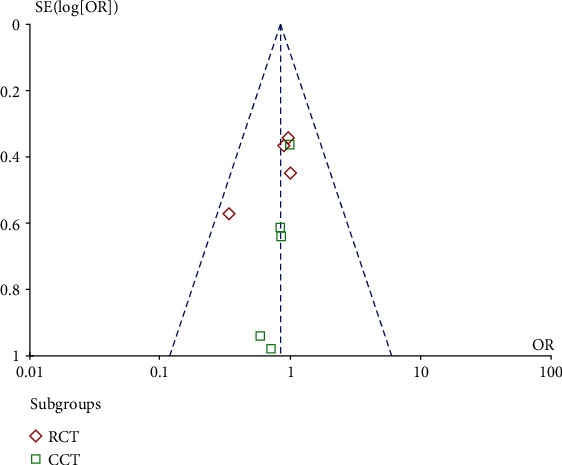
Funnel plot of the literature data on postoperative pancreatic fistula (POPF).

We drew funnel plots based on the POPF data. The results showed that the 95% CI data were similar among the studies, which were distributed in a roughly symmetrical manner between the two sides of the midline, suggesting that the results were unaffected by publication bias and were thus highly reliable (Figure 13).

## 4. Discussion

PD is a classic operation for benign and malignant lesions around the head of the pancreas. The incidence and complexity of postoperative complications are high due to the loss of organs and tissues [2, 21, 22]. DGE is a common complication after PD [23, 24]. Among the factors affecting DGE, the method used to reconstruct the digestive tract has been controversial. The use of Billroth II or Roux-en-Y reconstruction following PD to prevent DGE is also controversial [5–8, 13–17]. Meta-analyses that investigated the two reconstruction methods could not reach an agreement regarding which was superior [9, 10].

Consequently, we conducted this meta-analysis to analyze the characteristics of the two reconstruction methods and provide evidence-based guidance for clinical work. The results showed that traditional Billroth II reconstruction shortened the operation time compared to Roux-en-Y reconstruction, but no significant differences in any other complications were observed. Our results also indicated no significant differences between Roux-en-Y reconstruction and Billroth II reconstruction in DGE, A-grade DGE, B-grade DGE, or C-grade DGE (OR = 1.01, 95% CI: 0.50–2.03, *P* = 0.98; OR = 0.49, 95% CI: 0.17–1.45, *P* = 0.20; OR = 0.63, 95% CI: 0.29–1.38, *P* = 0.25; and OR = 2.13, 95% CI: 0.38–11.99, *P* = 0.39). Furthermore, no differences were detected in POPF, abscess, bile leak, infection, postoperative bleeding, or postoperative hospital stay (*P* > 0.05).

In 2015, Yang et al. [9] included three high-quality RCTs in their meta-analysis and reported that Billroth II reconstruction lowered the incidence of B- and C-grade DGE, although the small number of included studies was a limitation. Li et al. [10] conducted a case-control study that included 43 patients undergoing Roux-en-Y and 43 patients undergoing traditional Billroth II reconstruction after PD. The operation time of the Roux-en-Y group was longer than that of the traditional reconstruction group. A subsequent systematic review evaluated a series of postoperative complications. Klaiber et al. [25] systematically evaluated the postoperative complications of the two methods, and the results were largely consistent with those of the present study. However, it remains to be determined whether carrying out pancreatogastrostomy after PD had an impact on the overall results [26].

Based on previous studies, our study systematically screened the literature for studies of PD and excluded those in which PD was followed by pancreatogastrostomy. Articles with high heterogeneity were also excluded, so our analyses were characterized by relatively high homogeneity. We conducted this new systematic evaluation to overcome the deficiencies of previous research, and the results are reliable. However, this study also had some shortcomings. Although homogeneity was high, the number of included studies was small; hence, a larger study is needed.

We performed subgroup and sensitivity analyses of the outcomes with high heterogeneity; the results remained unchanged, except for operation time, further indicating high reliability. We also performed a subgroup analysis of operation time based on the RCTs and CCTs, and the results indicated that study type was the primary influencing factor. Therefore, an RCT or CCT with a large sample size is needed to further compare the operation times of Roux-en-Y and Billroth II digestive tract reconstruction.

In conclusion, we demonstrated that Roux-en-Y reconstruction took longer than Billroth II reconstruction after PD. However, complications were not different between the two reconstruction types. Therefore, it is suggested that consideration of the difference in operation time and patients' condition is needed to ensure that a suitable personalized surgical plan is implemented.

## Figures and Tables

**Table 1:** Basic parameters of the included studies.

Study	Publication time	Number in study	R/B (*n*)	Age (years)	Gender (M/F)	Study period	Study type	Nation
				Roux-en-Y/Billroth II				
Shimoda et al. [5]	2013	101	49/52	65.7 ± 11.1/66.5 ± 9.8	60/41	2008–2011	RCT	Japan
Busquets et al. [6]	2018	80	40/40	68.1 ± 11.7/65.6 ± 10.9	48/32	2013–2015	RCT	Spain
Tani et al. [7]	2014	151	75/76	69.6 ± 7.9/68.0 ± 8.9	81/70	2009–2012	RCT	Japan
Ke et al. [8]	2013	216	107/109	58.3 ± 5.9/59.3 ± 6.6	101/115	2006–2012	RCT	China
Perwaiz et al. [13]	2009	108	53/55	53.3 ± 12.1/53.5 ± 10.1	81/27	2003–2007	CCT	India
Kaman et al. [14]	2008	111	60/51	51 ± 13.3/50 ± 13.6	74/37	1994–2006	CCT	India
Ballas et al. [15]	2010	88	46/42	64.4 ± 9.5/60.9 ± 11.5	52/36	1994–2006	CCT	Greece
Ben-Ishay et al. [16]	2019	179	52/127	68.2 ± 9.6/68 ± 13.7	88/91	2010–2016	CCT	Israel
Casadei et al. [17]	2008	38	18/20	65.7 ± 10.0/56.3 ± 11.0	24/14	2006–2007	CCT	Italy

R: Roux-en-Y; B: Billroth II; M: male; F: female.

**Table 2:** Pathologies, surgery-related parameters, and quality scores.

Study	Pathologies (R/B)					PJ & GJ	Stenting (yes/no)	Definition of DGE	Definition of POPF	QS
	PL	BC	AC	DC	Other					
Shimoda et al. [5]						Duct-to-mucosa, end-to-side	Yes	ISGPS2007	ISGPF2005	5
Busquets et al. [6]	28/28	5/3	6/9	1/0		Duct-to-mucosa, end-to-side		ISGPS2007	ISGPF2005	5
Tani et al. [7]	55/57	11/11	7/6	2/2		Duct-to-mucosa, end-to-side	Yes	ISGPS2007	ISGPF2005	5
Ke et al. [8]	51/50	32/35	18/16	6/8		Duct-to-mucosa, end-to-side	Yes	Johns Hopkins [18]	Johns Hopkins [19], ISGPF2005	5
Perwaiz et al. [13]	12/13	10/4	24/25	3/6	4/7	Duct-to-mucosa, end-to-side	Yes	van Berge Henegouwen [20]	ISGPF2005	8
Kaman et al. [14]	30/24	9/4	16/18	5/5		Mucosa-to-mucosa, end-to-side	Yes	Self-definition	Self-definition	8
Ballas et al. [15]	59^∗^	8^∗^	15^∗^	6^∗^		Duct-to-mucosa, end-to-side	Yes	—	ISGPF2005	6
Ben-Ishay et al. [16]						End-to-side		ISGPS2007	—	8
Casadei et al. [17]	12/20	1/0	5/0			Duct-to-mucosa, end-to-side	Yes	—	ISGPF2005	7

R: Roux-en-Y; B: Billroth II; PL: pancreatic lesions; BC: biliary cancer; AC: ampullary cancer; DC: duodenal cancer; PJ: pancreatojejunostomy; GJ: gastrojejunostomy; QS: quality score; ISGPS: International Study Group of Pancreatic Surgery; ISGPF: International Study Group for Pancreatic Fistula; —: not mentioned. ^∗^total number (R+B).

## Data Availability

The datasets generated or analyzed during the current study are available from the corresponding author on reasonable request.

## References

[1] Rahib L., Smith B. D., Aizenberg R., Rosenzweig A. B., Fleshman J. M., Matrisian L. M. (2014). Projecting cancer incidence and deaths to 2030: the unexpected burden of thyroid, liver, and pancreas cancers in the United States. *Cancer Research*.

[2] Whipple A. O., Parsons W. B., Mullins C. R. (1935). Treatment of carcinoma of the ampulla of Vater. *Annals of Surgery*.

[3] Panwar R., Pal S. (2017). The International Study Group of Pancreatic Surgery definition of delayed gastric emptying and the effects of various surgical modifications on the occurrence of delayed gastric emptying after pancreatoduodenectomy. *Hepatobiliary & Pancreatic Diseases International*.

[4] Machado M. C., Cunha J. E., Bacchella T., Bove P. (1976). A modified technique for the reconstruction of the alimentary tract after pancreatoduodenectomy. *Surgery, Gynecology& Obstetrics*.

[5] Shimoda M., Kubota K., Katoh M., Kita J. (2013). Effect of Billroth II or Roux-en-Y reconstruction for the gastrojejunostomy on delayed gastric emptying after pancreaticoduodenectomy. *Annals of Surgery*.

[6] Busquets J., Martín S., Fabregat J., Secanella L., Pelaez N., Ramos E. (2019). Randomized trial of two types of gastrojejunostomy after pancreatoduodenectomy and risk of delayed gastric emptying (PAUDA trial). *The British Journal of Surgery*.

[7] Tani M., Kawai M., Hirono S. (2014). Randomized clinical trial of isolated Roux-en-Y versus conventional reconstruction after pancreaticoduodenectomy. *The British Journal of Surgery*.

[8] Ke S., Ding X.-m., Gao J. (2013). A prospective, randomized trial of Roux-en-Y reconstruction with isolated pancreatic drainage versus conventional loop reconstruction after pancreaticoduodenectomy. *Surgery*.

[9] Yang J., Wang C., Huang Q. (2015). Effect of Billroth II or Roux-en-Y reconstruction for the gastrojejunostomy after pancreaticoduodenectomy: meta-analysis of randomized controlled trials. *Journal of Gastrointestinal Surgery*.

[10] Li D.-B., Chai C., Cao L., Zhou Y.-M. (2017). Isolated Roux-en-Y reconstruction _versus_ conventional reconstruction after pancreaticoduodenectomy. *The Surgeon*.

[11] Jadad A. R., Moore R. A., Carroll D. (1996). Assessing the quality of reports of randomized clinical trials: is blinding necessary?. *Controlled Clinical Trials*.

[12] Wells G. A., Shea B., O’Conneell D., Peterson J., Welch V. (2011). The Newcastle-Ottawa Scale (NOS) for assessing the quality if nonrandomized studies in meta-analyses. http://www.ohri.ca/programs/clinical_epidemiology/oxford.htm.

[13] Perwaiz A., Singhal D., Singh A., Chaudhary A. (2009). Is isolated Roux loop pancreaticojejunostomy superior to conventional reconstruction in pancreaticoduodenectomy?. *HPB*.

[14] Kaman L., Sanyal S., Behera A., Singh R., Katariya R. N. (2008). Isolated Roux loop pancreaticojejunostomy vs single loop pancreaticojejunostomy after pancreaticoduodenectomy. *International Journal of Surgery*.

[15] Ballas K., Symeonidis N., Rafailids S. (2010). Use of isolated Roux loop for pancreaticojejunostomy reconstruction after pancreaticoduodenectomy. *World Journal of Gastroenterology*.

[16] Ben-Ishay O., Zhaya R. A., Kluger Y. (2019). Dual loop (Roux en Y) reconstruction with isolated gastric limb reduces delayed gastric emptying after pancreatico-duodenectomy. *World Journal of Gastrointestional Surgery*.

[17] Casadei R., Zanini N., Pezzilli R. (2008). Reconstruction after pancreaticoduodenectomy: isolated Roux loop pancreatic anastomosis. *Chirurgia Italiana*.

[18] Yeo C. J., Barry M. K., Sauter P. K. (1993). Erythromycin accelerates gastric emptying after pancreaticoduodenectomy. *Annals of Surgery*.

[19] Yeo C. J., Cameron J. L., Maher M. M. (1995). A prospective randomized trial of pancreaticogastrostomy versus pancreaticojejunostomy after pancreaticoduodenectomy. *Annals of Surgery*.

[20] van Berge Henegouwen M. I., Van Gulik T. M., DeWit L. T. (1997). Delayed gastric emptying after standard pancreaticoduodenectomy versus pylorus-preserving pancreaticoduodenectomy: an analysis of 200 consecutive patients. *Journal of American College of Surgeons*.

[21] DeOliveira M. L., Winter J. M., Schafer M. (2006). Assessment of complications after pancreatic surgery. *Annals of Surgery*.

[22] van Hilst J., de Rooij T., Bosscha K. (2019). Laparoscopic versus open pancreatoduodenectomy for pancreatic or periampullary tumours (LEOPARD-2): a multicentre, patient-blinded, randomised controlled phase 2/3 trial. *The Lancet Gastroenterology & Hepatology*.

[23] Kurahara H., Shinchi H., Maemura K. (2011). Delayed gastric emptying after pancreatoduodenectomy. *The Journal of Surgical Research*.

[24] Glowka T. R., Webler M., Matthaei H. (2017). Delayed gastric emptying following pancreatoduodenectomy with alimentary reconstruction according to Roux-en-Y or Billroth-II. *BMC Surgery*.

[25] Klaiber U., Probst P., Knebel P. (2015). Meta-analysis of complication rates for single-loop versus dual-loop (Roux-en-Y) with isolated pancreaticojejunostomy reconstruction after pancreaticoduodenectomy. *The British Journal of Surgery*.

[26] el Nakeeb A., Hamdy E., Sultan A. M. (2014). Isolated Roux loop pancreaticojejunostomy versus pancreaticogastrostomy after pancreaticoduodenectomy: a prospective randomized study. *HPB*.

